# Low-dose IL-2 mitigates glucocorticoid-induced Treg impairment and promotes improvement of SLE

**DOI:** 10.1038/s41392-023-01350-6

**Published:** 2023-04-03

**Authors:** Haotian Zhou, Xiaozhen Zhao, Ruijun Zhang, Miao Miao, Wenwen Pei, Zijun Li, Yimin Li, Jing He, Zhanguo Li, Xiaolin Sun

**Affiliations:** 1 Department of Rheumatology and Immunology, Clinical Immunology Center, Beijing Key Laboratory for Rheumatism Mechanism and Immune Diagnosis (BZ0135), Peking University People’s Hospital, No.11 Xizhimen South Street, 100044 Beijing, China; 2 Department of Rheumatology, Beijing Children’s Hospital, Capital Medical University, National Centre for Children’s Health, 100045 Beijing, China; 3 Peking-Tsinghua Center for Life Science, Beijing, China

**Keywords:** Lymphocytes, Immunological disorders

**Dear Editor**,

Previous studies have proved that regulatory T cell (Treg) insufficiency contributed to the development of autoimmune conditions including systemic lupus erythematosus (SLE). Conventional immunosuppressive treatment was reported to downregulate beneficial Tregs together with pathogenic effector immune cells, which may impede a rapid achievement of optimal therapeutic effects.^1,2^ As the most effective and widely applied immunosuppressive medication for SLE, glucocorticoid treatment has the risk of excessive suppression of immune cell subsets. There is a discrepancy in the influence of glucocorticoids on regulatory T cells (Treg).^3,4^ Therefore, in this study, we compared the effects of prednisone treatment with and without low-dose IL-2 supplementation on Tregs in SLE patients to identify the influence of glucocorticoid on Tregs and to elucidate whether low-dose IL-2 could reverse the impact of glucocorticoid on Tregs, thereby improve the therapeutic effects in SLE.

With prednisone treatment without low-dose IL-2, Treg proportions in the peripheral blood decreased in 36.36% of the patients (4/11), while no Treg decrease occurred in patients treated by prednisone combined with 1 million IU rhIL-2 administered subcutaneously every other day (0/14). The treatment period lasted for two weeks. With low-dose IL-2 supplementation, 92.86% of the patients showed obvious increases in Treg levels. In comparison, when treated with prednisone without IL-2 supplementation, only 27.27% of these patients had increased Treg ratio (Fig. 1a). We also observed significant increases of absolute numbers of Treg cells after prednisone plus low-dose IL-2 treatment, while treatment solely with prednisone induced a significant decrease of Treg absolute numbers (Supplementary Fig. 1). We further characterized the alteration of Treg sub-populations defined by CD25 and CD45RA expression^5^ (Fig. 1b). The proportions of total Treg (CD4^+^CD25^hi^CD127^low^), active Treg (CD4^+^CD127^low^CD45RA^-^CD25^+++^) and resting Treg (CD4^+^CD127^low^CD45RA^+^CD25^+++^) in SLE patients treated by prednisone together with low-dose IL-2 all significantly increased, while there was no significant difference in prednisone group (Fig. 1a, *P* = 0.033 vs *P* = 0.811; Fig. 1c, *P* = 0.042 vs *P* = 0.463; Fig. 1d, *P* = 0.006 vs *P* = 0.688). Actually, total Treg, active Treg, and resting Treg were decreased in most cases of the prednisone group (Fig. 1b–d). The overall CD25 (IL-2 receptor alpha) expression in Treg cells were also significantly enhanced in all patients in the IL-2 combination group. By contrast, 36.36% (4/11) of patients treated with prednisone alone showed decreased CD25 levels on their Tregs (Fig. 1e). The expression level of CD25 correlates with the activation of IL-2 signaling and Tregs,^5^ and this result suggests that prednisone treatment might decrease Treg activity in a proportion of patients, while low-dose IL-2 supplementation to patients treated by prednisone effectively restored Treg activity in vivo (Fig. 1e). A group of patients with reduced Tregs after prednisone treatment were further treated with the addition of low-dose IL-2 on unchanged prednisone treatment. The result showed that low-dose IL-2 supplementation reversed glucocorticoid-induced Treg decrease, and further confirmed that low-dose IL-2 supplementation mitigated glucocorticoid-induced Treg impairment (Fig. 1f).

**Fig. 1:**
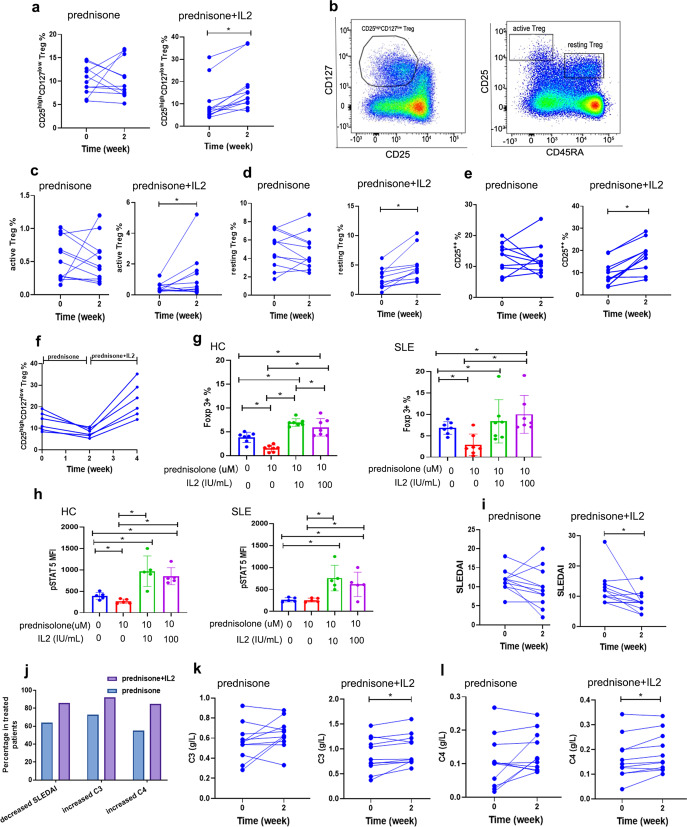
Differential effects of prednisone with or without IL-2 supplementation on Treg subsets and disease improvement in SLE patients. **a** Shows the percentages of CD25^high^CD127^low^ Treg before and after 2 weeks of treatment. **b** Treg were gated as CD25^high^CD127^low^ in CD4^+^ T cells. Treg subsets were defined based on the expression of CD45RA and CD25: CD45RA^-^CD25^+++^ active Treg, CD45RA^+^CD25^++^ resting Treg. **c**, **d** Show the percentages of active Treg and resting Treg before and after 2 weeks of treatment. **e** Shows the expression of CD25 before and after 2 weeks of treatment. **f** Shows the reduced Treg levels in patients treated with prednisone alone were rescued by the addition of low-dose IL-2. **g**, **h** Show that IL-2 restored prednisolone-induced decrease on expression of (**g**) Foxp3 and (**h**) pSTAT5 in vitro. **j** The percentages of patients with decreased SLEDAI and increased C3 and C4 was compared between prednisone and prednisone+IL-2. **i** SLEDAI score, levels of (**k**) C3 and (**l**) C4 of SLE patients before and after 2 week treatment with prednisone with/without IL-2 are shown

In vitro treatment of Treg cells solely with prednisolone, the bio-active product of prednisone in vivo, significantly impaired multiple key moleculars in Treg differentiation and function. The supplementation of low-dose IL-2 to prednisolone treatment showed a protective role on these moleculars. Prednisolone treatment down-regulated the expression of Foxp3 in CD4 + T cells, and IL-2 significantly reversed the prednisolone-induced decrease in Foxp3 expression in both CD4^+^ T cells from SLE patients and healthy donors (Fig. 1g, *P* < 0.05). CD4^+^Foxp3^+^ Treg cells express CD39, CTLA-4, and ICOS, which are effector moleculars involved in their immunosuppressive function.^6,7^ The expression of Bcl-2 is critical to maintain Treg survival. It was found that prednisolone significantly decreased the expression of CD39 in CD4^+^Foxp3^+^ Treg cells, while IL-2 significantly reversed the effect of prednisolone (Supplementary Fig. 2a, *P* < 0.05). Similarly, IL-2 supplementation reversed the downregulation effect of prednisolone on the expression of ICOS in Treg cells in both healthy donors and SLE patients (Supplementary Fig. 2b). However, the upregulation of ICOS in Tregs from SLE patients did not reach statistical significance, which was consistent with the report that Tregs in SLE patients were less sensitive to IL-2 than those from healthy people^8^ (Supplementary Fig. 2b). The expression of Bcl-2 in Treg cells could also be up-regulated by IL-2 in SLE patients and healthy controls (Supplementary Fig. 2c, *P* < 0.05). Unlike CD39 and ICOS, CTLA-4 expression was not affected by prednisolone or IL-2 (Supplementary Fig. 2d).

The expression of Foxp3 and these effector molecules were driven by the activation of STAT5. Our study showed that prednisolone treatment-induced a decrease of STAT5 phosphorylation in Tregs from both SLE patients and healthy donors, while prednisolone plus IL-2 significantly increased the levels of pSTAT5 (Fig. 1h).

The above results showed that low-dose IL-2 supplementation to prednisone treatment was beneficial for Treg cells, which might be helpful to curb autoimmunity. To determine if there is any clinical benefit of low-dose IL-2 supplementation for prednisone treatment, we compared the changes of clinical parameters of SLE patients treated with prednisone alone and prednisone combined with low-dose IL-2. After 2 weeks of treatment, we found SLEDAI decreased more significantly in the prednisone plus low-dose IL-2 group (Fig. 1i). SLEDAI decreased in 85.71% of the patients treated with combination of prednisone and low-dose IL-2, while only 63.64% of patients treated with only prednisone showed decreased SLEDAI (Fig. 1j). The IL-2 combination group also showed more significant elevation of C3 and C4 (Fig. 1k, l). Higher percentages of patients with C3 or C4 elevation were observed in the prednisone plus IL-2 group (Fig. 1j). More statistically significant improvements in rash and fever were also observed in patients with IL-2 combination treatment (Supplementary Table 1).

Our study have several limitations. First, the mechanism of how prednisone impair Tregs are not studied, which needs to be verified by further experiments. Second, the cohort size and treatment duration are limited, since SLE needs long-term treatment and SLE medication effects often last for a short period, the evaluation of the long-term efficacy of prednisone plus IL-2 treatment in a larger cohort size is necessary in our future study. Another limitation of this study is we did not evaluate the changes of effector moleculars and transcription factors sustaining Treg homeostasis directly from treated patients due to limitations of sampling and clinical tests. These in vitro results should be replicated in treated patients in future studies.

The mechanism of glucocorticoid treatment reducing Treg cells might be attributed to the downregulation of levels of CD25, Foxp3 and pSTAT5 levels by glucocorticoid. Foxp3 and STAT5 are both transcription factors indispensable in Treg development, differentiation and phenotype maintenance. Decrease of Foxp3 expression and STAT5 activation by prednisone treatment may impair Treg differentiation and phenotype sustaining. IL-2-IL-2 receptor interaction is also necessary to maintain the Treg normal functions by inducing downstream STAT5 activation and Foxp3 expression, which in turn further promotes the transcription of IL-2 receptor subunits like CD25 and intensifying IL-2 signaling. Therefore, the decrease of CD25 expression in Treg cells induced by prednisone treatment might also disrupt the positive feedback loop of IL-2 signaling of Tregs, and lead to the decrease of Treg cells.

In conclusion, our study demonstrates that low-dose IL-2 might mitigate glucocorticoid-induced Treg impairment, which might promote a more rapid improvement of SLE.

## Supplementary information

Supplementary Fig or Table

## Data Availability

The data that support the findings of this study are available from the corresponding authors.
