# Supplementary material for: Low-dose IL-2 mitigates glucocorticoid-induced Treg impairment and promotes improvement of SLE
Source: Signal Transduct Target Ther. 2023 Apr 3;8:141. doi: 10.1038/s41392-023-01350-6 (PMC10068597; doi:10.1038/s41392-023-01350-6)
Supplement: Supplementary file 1 — Supplementary Fig or Table [file 41392_2023_1350_MOESM1_ESM.docx]

Supplementary Fig or Table for

Low-dose IL-2 mitigates glucocorticoid induced Treg impairment and promotes improvement of SLE

Haotian Zhou^*^, Xiaozhen Zhao^*^, Ruijun Zhang, Miao Miao, Wenwen Pei, Zijun Li, Yimin Li, Jing He, Zhanguo Li^#^, Xiaolin Sun^#^

Correspondence to: sunxiaolin_sxl@126.com, li99@bjmu.edu.cn

**This PDF file includes:**

Materials and Methods

Supplementary Figs 1 to 3

Supplementary Tables 1 to 3

Materials and Methods

Patients

All SLE patients were diagnosed according to the 1997 revised classification criteria of the American College of Rheumatology ^1^. The background treatment with antimalarials and immunosuppressants was shown in online supplementary appendix Table S1, which were comparable between two groups. The background therapy was unable to control disease, and not changed after the subsequent addition of prednisone alone or prednisone plus low-dose IL-2 supplementation. Exclusion criteria included: active severe neuropsychiatric manifestations of SLE; history of treatment with rituximab or other biologics; severe comorbidities including heart failure (≥grade III New York Heart Association), renal insufficiency (creatinine clearance ≤30 mL/min) or hepatic insufficiency (alanine aminotransferase or aspartate aminotransferase ≥2 times of the upper limit of the normal range); active infection (hepatitis B or C virus, Epstein-Barr virus, HIV or Mycobacterium tuberculosis); history of chronic infection; malignancy; pregnancy or lactation in females. Age and sex matched healthy controls (HC) were also recruited from the same hospital according to their medical records. This study was approved by the Ethics Committee of Peking University People’s Hospital (Approval number 2014PHB087-04 and 2019PHB234-02). All participants signed the consent for this study.

Phenotyping of Treg cells by in vitro treatment of prednisolone and IL-2

Peripheral blood mononuclear cells (PBMCs) were isolated by Ficoll-Hypaque density gradient centrifugation. PBMCs from SLE patients or healthy donors were divided into 96-well flat-bottom cell culture plates and cultivated in complete RPMI 1640 medium supplemented with fetal bovine serum (10%) and penicillin and streptomycin (1%). Prednisolone (Sigma Aldrich) and different concentrations of IL-2 (Beijing SL Pharma) were added into different wells. The final concentration of prednisolone was 10uM, and IL-2 concentrations were 0, 10 and 100 IU/ml for different stimulation groups (Supplementary Fig 1). After 96 hours, the PBMCs were harvested and the Treg subset and its effecor moleculars and transcriptional factors were examined by flow cytometry.

Flow cytometry and intracellular cytokine staining assays

Antibodies used for immunophenotyping were listed in Supplementary Table 2. For immunophenotyping of patients receiving prednisone or prednisone plus low-dose IL-2, peripheral blood mononuclear cells (PBMCs) were isolated by Ficoll-Hypaque density gradient centrifugation. PBMCs were stained for 30 min in the dark at 4◦C with the monoclonal antibodies CD4, CD25, CD127 and CD45RA. Based on the surface markers ^2,3^, total Treg (CD4^+^CD25^hi^CD127^low^), active Treg (CD4^+^CD127^low^CD45RA^-^CD25^+++^) and resting Treg (CD4^+^CD127^low^CD45RA^+^CD25^+++^) were analyzed by flow cytometry using FACSAria II (BD) and FlowJo software (Tree Star). Tfh cell subset was defined as CD3^+^CD4^+^CXCR5^+^PD1^high^CCR7^low 4^, Th17 cells as CD3^+^CD4^+^CXCR3^-^CCR6^+^CCR7^low 5,6^, Th1 cells as CD3^+^CD4^+^CXCR3^+^CCR6^-^CCR7^low^ ^6^ and Th2 cells as CD3^+^CD4^+^CXCR3^-^CCR6^-^CCR7^low 6^.

For phenotype evaluation of Treg cells by in vitro treatment with prednisolone or prednosolone plus IL-2, the key transcription factors and effector moleculars of Tregs were also evaluated by flow cytometry. Briefly, after stimulation, the cell surface markers CD4, CD25, and CD127 were stained with the fluorescently labelled monoclonal antibodies mentioned above. Next, the cells were fixed and permeabilised for 30 min at room temperature at dark with the Transcription Factor Fixation/Permeabilisation Concentrate and Diluent buffer set (Invitrogen). Then the cells were stained with fluorescence labelled monoclonal antibodies against Foxp3, pSTAT5, Bcl-2, CD39, CTLA-4 and ICOS. Antibodies used for the Treg phenotyping were listed in Supplementary Table 3.

Statistics

All data were presented as the median (interquartile range) unless otherwise stated. The Mann–Whitney U-test, Chi-square (χ2) test and T test were used as appropriate. A P-value<0.05 was considered statistically significant. Data were analyzed using the SPSS statistical software package (version 24.0, IBM) and GraphPad Prism (Version 7.0, Graph Pad Software).

References

1 Hochberg, M. C. Updating the American College of Rheumatology revised criteria for the classification of systemic lupus erythematosus. *Arthritis Rheum*. **40**, 1725, (1997).

2 Miyara, M. *et al.* Functional delineation and differentiation dynamics of human CD4+ T cells expressing the FoxP3 transcription factor. *Immunity*. **30**, 899-911, (2009).

3 Seddiki, N. *et al.* Expression of interleukin (IL)-2 and IL-7 receptors discriminates between human regulatory and activated T cells. *J Exp Med*. **203**, 1693-1700, (2006).

4 He, J. *et al.* Circulating precursor CCR7(lo)PD-1(hi) CXCR5(+) CD4(+) T cells indicate Tfh cell activity and promote antibody responses upon antigen reexposure. *Immunity*. **39**, 770-781, (2013).

5 Acosta-Rodriguez, E. V. *et al.* Surface phenotype and antigenic specificity of human interleukin 17-producing T helper memory cells. *Nat Immunol*. **8**, 639-646, (2007).

6 Miao, M. *et al.* Therapeutic potential of targeting Tfr/Tfh cell balance by low-dose-IL-2 in active SLE: a post hoc analysis from a double-blind RCT study. *Arthritis Res Ther*. **23**, 167, (2021).


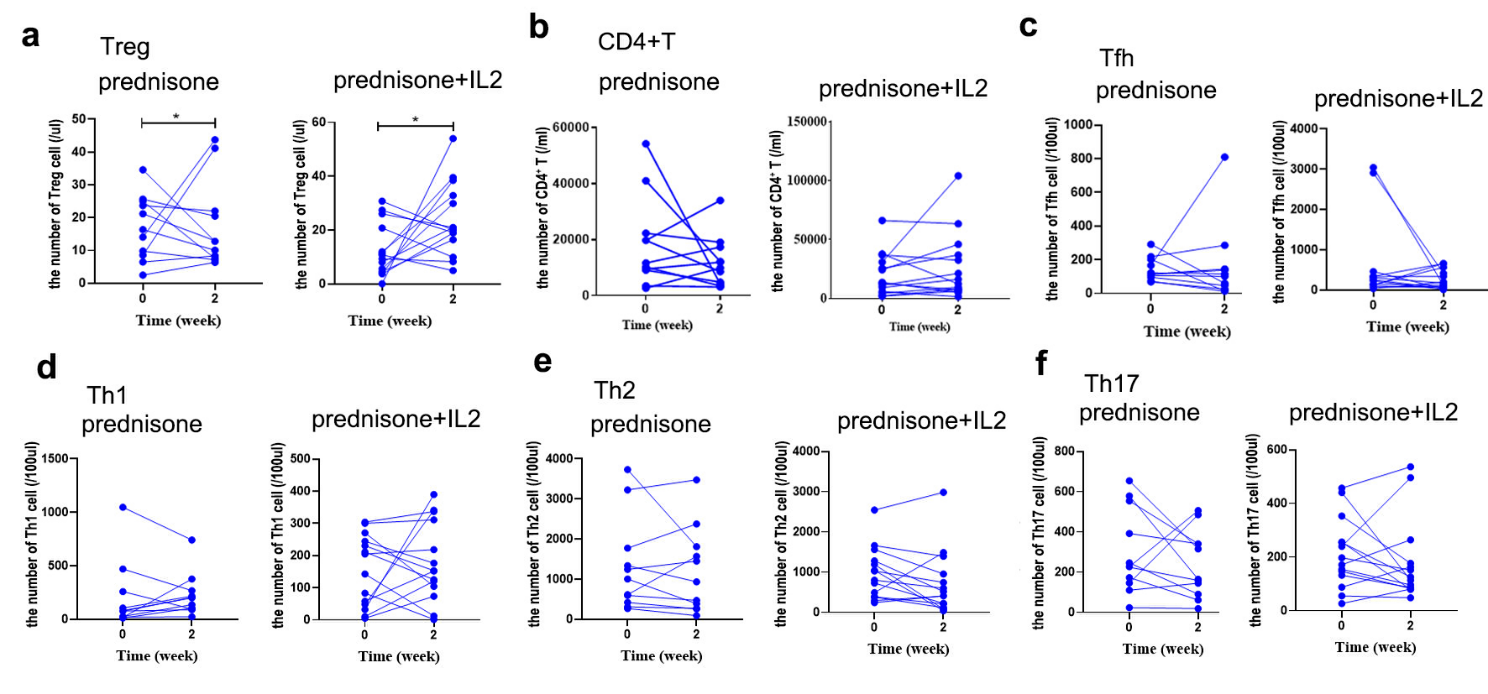
Supplementary Fig 1.

Supplementary Fig 1. Different effects between IL-2 and prednisone on T cell subsets in vivo in SLE patients. The absolute number of Treg(a), CD4+ T(b), Tfh(c), Th1(d), Th2(e), Th17(f).

Supplementary Fig 2.


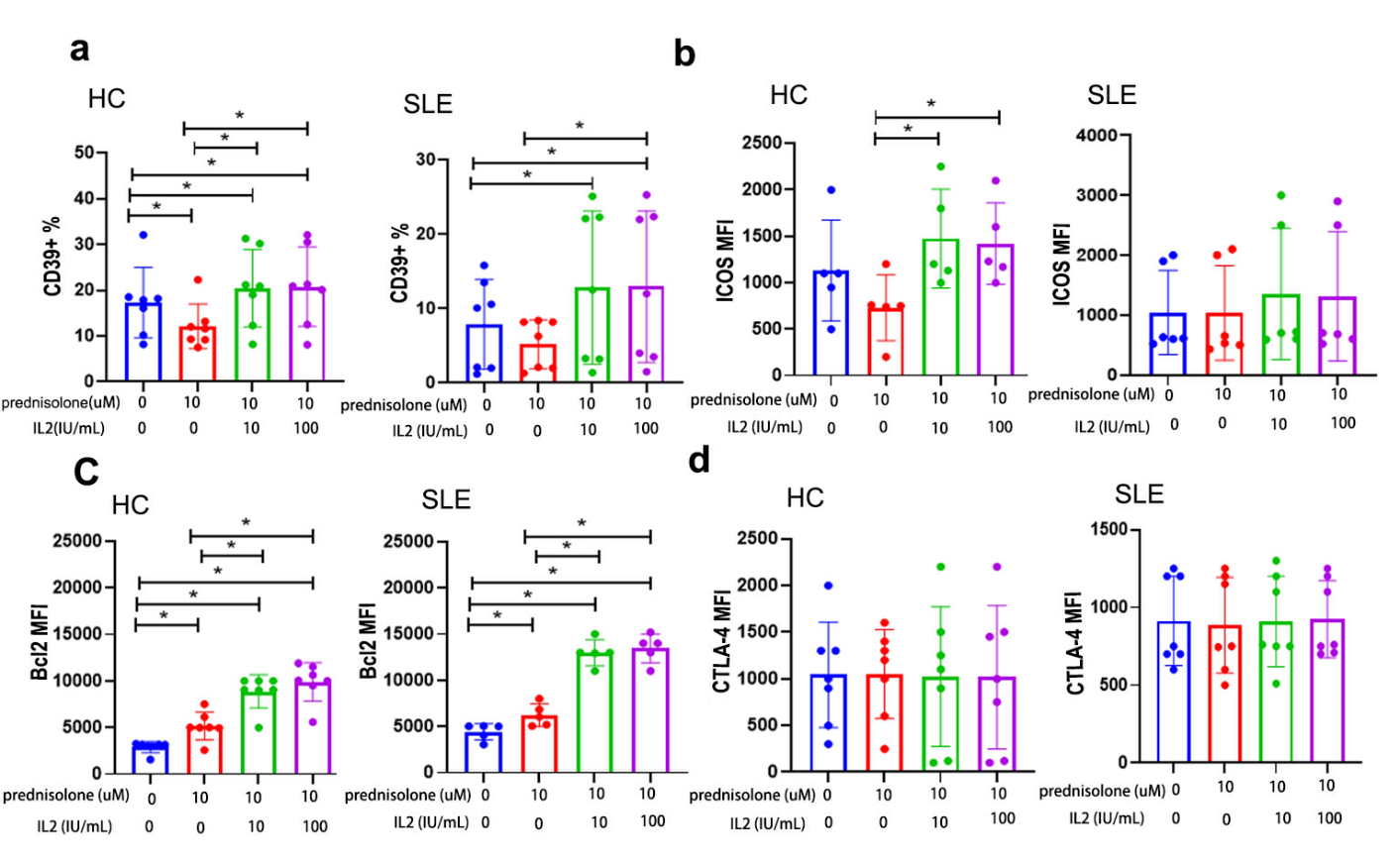


Supplementary Fig 2. Low-dose IL-2 supplementation to prednisolone treatment showed a protective role on key moleculars involved in Treg differentiation and function. PBMCs from SLE patients and healthy donors were cultured with prednisolone with/without IL-2 for 4 days. (a-c) IL-2 restored prednisolone induced decrease on expression of (a) CD39, (b) ICOS and (c) Bcl-2. CTLA-4 expression (d) was not affected by prednisolone or IL-2.

Supplementary Fig 3.

**

**


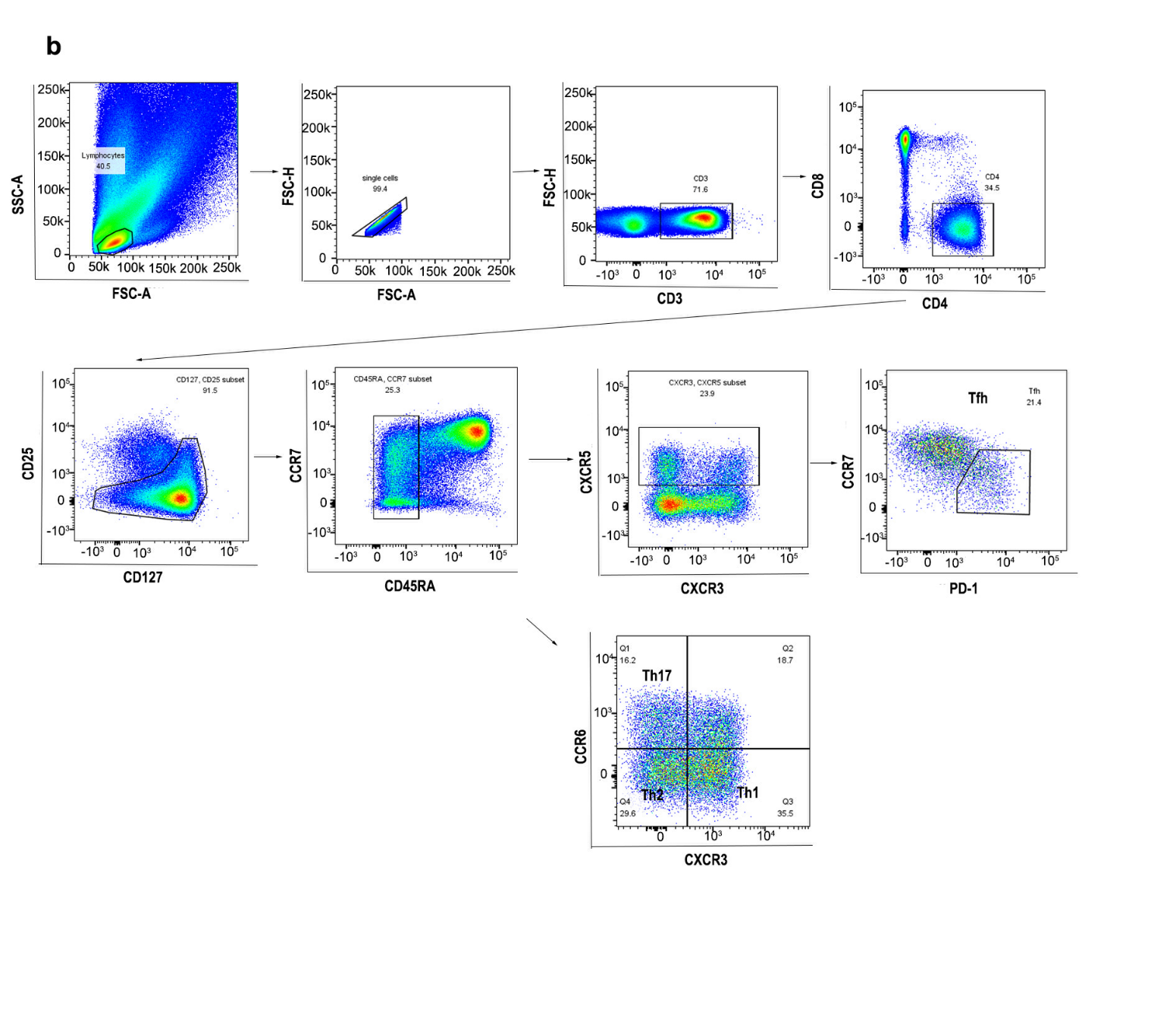


Supplementary Fig 3. The gating strategies of Treg, active Treg, resting Treg(a),Tfh,Th1,Th2, and Th17(b).

Supplementary Table 1.

Responses of SLE patients to low-dose IL-2 treatment.

| Characteristics | Baseline | Week 2 | P value |
| --- | --- | --- | --- |
| SLEDAI, median(range) |  |  |  |
| pred+IL-2 | 12.64(8-27) | 8.78 (4-18) | **0.024** |
| pred | 11.73(6-18) | 9.73(2-16) | 0.282 |
| Rash, n (%) |  |  |  |
| pred+IL-2 | 5 (35.71) | 1 (7.14) | **0.045** |
| pred | 4 (36.36) | 1 (9.09) | 0.127 |
| Oral ulceration, n (%) |  |  |  |
| pred+IL-2 | 1 (7.14) | 0 (0) | 0.309 |
| pred | 0 (0) | 0 (0) | - |
| Arthritis, n (%) |  |  |  |
| pred+IL-2 | 2 (14.29) | 0 (0) | 0.142 |
| pred | 3 (27.27) | 1 (9.09) | 0.280 |
| Vasculitis, n (%) |  |  |  |
| pred+IL-2 | 1 (7.14) | 0 (0) | 1.000 |
| pred | 1 (9.09) | 0 (0) | 0.306 |
| Alopecia, n (%) |  |  |  |
| pred+IL-2 | 2 (14.29) | 0 (0) | 0.142 |
| pred | 3 (27.27) | 1 (9.09) | 0.269 |
| Fever, n (%) |  |  |  |
| pred+IL-2 | 5 (35.71) | 0 (0) | **0.014** |
| pred | 2 (18.18) | 0 (0) | 0.138 |
| Myositis, n (%) |  |  |  |
| pred+IL-2 | 1 (7.14) | 0 (0) | 0.306 |
| pred | 1 (9.09) | 0 (0) | 0.306 |
| Anti-ds-DNA, IU/mL,  median (range) |  |  |  |
| pred+IL-2 | 87.33 (1-465.78) | 68.67 (1-424.51) | 0.708 |
| pred | 526.41 (1-2525.53) | 445.56 (1-2152.7) | 0.838 |

Supplementary Table 2.

Baseline characteristic of SLE patients (n=25).

| Characteristics | IL-2 (n=14) | Pred (n=11) | P value |
| --- | --- | --- | --- |
| Age, year, mean±SD | 30.93±9.31 | 26.73±4.08 | 0.178 |
| Female/Male | 13/1 | 11/0 | 0.366 |
| Weight, kg, mean±SD | 51.55±6.21 | 58.61±8.72 | 0.038 |
| Height, cm, mean ±SD | 161.31±6.30 | 162.00±5.92 | 0.798 |
| Duration, month, mean ±SD | 43.00±61.23 | 71.82±63.95 | 0.264 |
| SLEDAI, median (range) | 12.64±4.91 | 11.73±2.90 | 0.590 |
| Medications | | | |
| HCQ | 14(100) | 11(100) | 1.000 |
| CTX | 2(14.29) | 0(0) | 0.191 |
| AZA | 1(7.14) | 1(9.09) | 0.859 |
| Prednisone | 14(100) | 11(100) | 1.000 |
| CsA | 0(0) | 1(9.09) | 0.250 |
| MMF | 3(21.43) | 4(36.36) | 0.409 |
| LEF | 2(14.29) | 0(0) | 0.191 |

Pred: prednisone; HCQ: hydroxychloroquine; CTX: cyclophosphamide; MMF: Mycophenolate Mofetil; LEF: leflunomide; AZA: azathioprine; CsA: cyclosporin A.

Supplementary Table 3.

Antibodies used in flow cytometric analysis in this study.

| Target antigen | Clone | Fluorochrome | Vendor |
| --- | --- | --- | --- |
| CD4 | SK3 | FITC | Biolegend |
| CD25 | BC96 | PE | Biolegend |
| CD127 | A019D5 | Brilliant Violet 605 | Biolegend |
| CD45RA | HI100 | Brilliant Violet 510 | Biolegend |
| CD39 | TU66 | Brilliant Violet 421 | BD Horizon |
| CTLA-4 | BNI3 | APC | BD Horizon |
| ICOS | 2D3/B7-H2 | PECF594 | BD Horizon |
| Bcl-2 | N46-467 | Alexa Fluor 647 | BD Horizon |
| Foxp3 | 259D/C7 | Alexa Fluor 647 | BD Horizon |
| Foxp3 | 259D/C7 | PE | BD Horizon |
| pSTAT5 | C71E5 | Alexa Fluor 647 | Cell Signaling technology |
